# How to Assess the Existence of Competing Strategies in Cognitive Tasks: A Primer on the Fixed-Point Property

**DOI:** 10.1371/journal.pone.0106113

**Published:** 2014-08-29

**Authors:** Leendert van Maanen, Ritske de Jong, Hedderik van Rijn

**Affiliations:** 1 Department of Psychology, University of Amsterdam, Amsterdam, The Netherlands; 2 Department of Psychology, University of Groningen, Groningen, The Netherlands; Universidad de Zarazoga, Spain

## Abstract

When multiple strategies can be used to solve a type of problem, the observed response time distributions are often mixtures of multiple underlying base distributions each representing one of these strategies. For the case of two possible strategies, the observed response time distributions obey the fixed-point property. That is, there exists one reaction time that has the same probability of being observed irrespective of the actual mixture proportion of each strategy. In this paper we discuss how to compute this fixed-point, and how to statistically assess the probability that indeed the observed response times are generated by two competing strategies. Accompanying this paper is a free R package that can be used to compute and test the presence or absence of the fixed-point property in response time data, allowing for easy to use tests of strategic behavior.

## Introduction

Almost all intentional behavior is the result of applying strategies to problems. Theorizing in cognitive psychology thus often involves the assertion that humans have access to a number of alternative strategies to solve a particular task, and that the observed behavior on a particular trial is the result of the execution of one particular strategy. On other trials an alternative strategy might have been selected, which may result in differences in the observed behavior. For example, idiomatic or fixed-phrase language processing is thought to be a dual-route process [1]. That is, idiomatic expressions (like *kick the bucket, or half past twelve*) are thought to be either retrieved in full from memory, or composed out of the constituent words when required. Whether the retrieval strategy or the production strategy is more likely for any given utterance depends on the frequency of the expression. However, it is difficult to find experimental support for a dual-route theory of idiomatic language processing, because the observed responses over a series of trials are a mixture of the two strategies. Thus, as it is unknown which strategy was used on which trial, the observed distribution of response times might as well be generated by a single strategy.

Many theoretical paradigms assume that behavior is the result of similar mixtures of processes (e.g., visual word recognition and reading aloud [2], task switching [3], visual working memory [4], exploration versus exploitation [5], speed-accuracy trade-off [6], the PRP effect [7], [8]). An important but often implicit property shared by these theoretical accounts is the assumption that the observed response time (RT) distribution is a mixture of two or more processing time distributions, representing the processing times of the possible strategies. This mixture assumption is often based on theoretical arguments as it is not straightforward to demonstrate the existence of multiple processing time distributions: That is, multimodality is difficult to assess. However, under certain constraints, most notably the constraint that the mixture is based on two distributions, this mixture assumption provides testable predictions, and this paper presents a simple method (and an R package) for testing those predictions. Given the constraints associated with the fixed-point property, the application of our method is limited to theories that assume two competing processes. Nevertheless, because of the proliferation of theories that assume two competing strategies, the work presented here provides important behavioral predictions and methods to test these predictions which can be used to falsify or support claims of competing strategies.

### The fixed-point property

**Figure 1:**
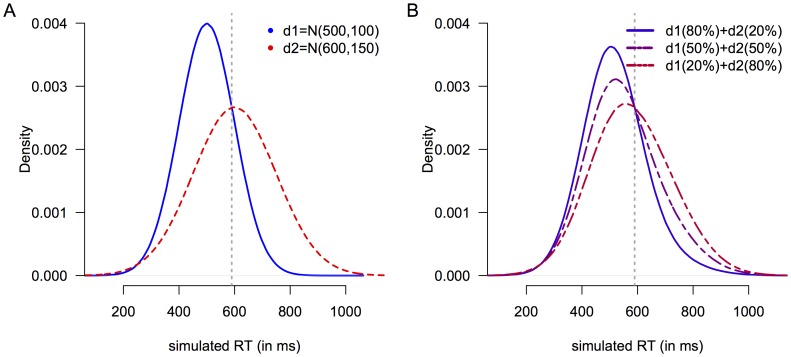
Illustration of the fixed-point property. Panel A shows density plots for two base distributions. The blue line reflects an RT distribution for Strategy 1 (d1), with a mean of 500 ms and a standard deviation of 100, the red line an RT distribution (d2) for Strategy 2 (mean = 600, SD = 150). Panel B shows three mixtures of the two base distributions with mixture proportions as indicated in the legend. The vertical line shown in both panels is drawn at the common coordinate or fixed-point at ∼590 ms.

An important property of a set of mixture distributions that are all based on the combination of two identical base distributions is the so-called fixed-point property [9]. The fixed-point property entails that the probability density functions of distributions with different mixture proportions share a common coordinate (Figure 1). Although the fixed-point property is present in all types of data, here we will focus on the case of response time distributions, and assume that the mixture consists of response times generated by one of two strategies (e.g., Strategy 1 or Strategy 2). The common coordinate means that independent of the relative proportion of Strategy 1 or Strategy 2 usage, there exists an RT that has the same probability of occurring irrespective of the actual relative proportion.

Despite its wide applicability, there are only a few studies that discuss the fixed-point property (e.g, [10]–[14]), let alone formally test its presence (or absence) in the data (the exception being [10], [14], who tested for the *absence* of the fixed-point property). We can see two reasons that have precluded studying the fixed-point property in the past. The first reason is that computing the probability density of the observed response time distribution is not trivial. This can be seen by considering histograms, arguably the simplest method to summarize frequency distribution data. Despite its apparent simplicity, the exact shape of the histogram depends on the number of bins, or alternatively the bin size. That is, the frequency observed for each bin is a function of the number of bins, and obviously if the number of bins is 1, all observations are categorized to belong to this bin. As the fixed-point property entails that there exists a bin that has the same frequency for each mixture proportion, if just one bin is used, the fixed-point property holds for all mixture proportions with equal number of observations. While true, this case would be uninformative, as the location of the fixed point remains unknown. By contrast, if there would be a bin with equal number of observations across mixture proportions in a histogram with many bins, it would be very informative. Unfortunately, the probability that this happens decreases with the number of bins. Consequently, the probability of finding the fixed-point property depends on the choice of bin size.

The second reason that might have withheld researchers to use the fixed-point property is that performing a statistical test to support the presence of the fixed-point property requires supporting the null hypothesis (i.e., the frequency does *not* depend on mixture proportion for one bin) in a classical null hypothesis significance test framework, which is atypical. It is a well-known problem that even if classical test statistics do not reach significance, there may be reasons other than the similarity between the compared conditions, such as the power of the test (see e.g., [15]). A non-significant result can thus be never attributed to the null hypothesis. In the next section, we will reiterate the important properties of the fixed-point property, introduce our method for computing and testing it, and discuss both the issues raised above.

### Computing and testing the fixed-point property

The fixed-point property is a mathematical property of binary mixture distributions: The density function of a binary mixture distribution (*g(t)*) is a combination of two base distributions *f_1_(t)* and *f_2_(t)*, weighted by the mixture proportion *p*:
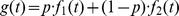
If *p* is 0 or 1, the mixture distribution is equal to one of the base distributions, i.e., *g_p = 0_(t)*  =  *f_2_(t)* or *g_p = 1_(t)*  =  *f_1_(t)*. If the two base distributions overlap, there is a time point *t_o_* such that 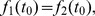
meaning that both densities are equal for *t_o_*. Combined these equations provide the fixed point property:
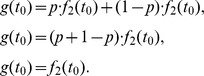



Thus, the density of the mixture at *t_0_*, *g(t_0_)*, does not depend on the mixture proportion *p* but is equal to the density of either base distribution at *t_0_* [9],[12]. This implies that RT distributions that consist of a mixture of two base distributions have a common RT with identical probability density, independent of the mixture proportion.

### Computing the fixed-point property

To infer the fixed-point property in experimental data, for example if one wants to assess whether two conditions just differ in terms of the relative proportions of Strategy 1 and Strategy 2 usage, the first step is to estimate the continuous density functions of the data. That is, for each condition – reflecting a mixture proportion – the empirical probability density has to be computed. Computing the density instead of a histogram solves in part the issue of the bin size discussed previously. A straightforward method for computing the continuous density function is kernel density estimation (e.g., [16]–[18]), which estimates the density of a set of data points (in our case, response times) by summing kernels that are centered on the data points. This method can be thought of as smoothing a histogram. Typically (but not necessarily), the kernels are normal distributions with a standard deviation *h* that depends on the number of data points [17]. The standard deviation of the kernel determines the degree of smoothness of the estimated density function (i.e., *h* can be seen as the smoothing factor). Selecting an appropriate *h* parameter is a procedure of some delicacy. If *h* is too small, than the estimated density may include local noise. If *h* is too large, potentially important properties of the distribution will also be smoothed out, such as multimodality [17]. However, there are a number of methods by which *h* can be set (e.g., [17], [19]–[21]). In the Simulations section below we will explore the extend to which the choice of *h* influences whether the fixed-point property is recovered from the data.

**Figure 2:**
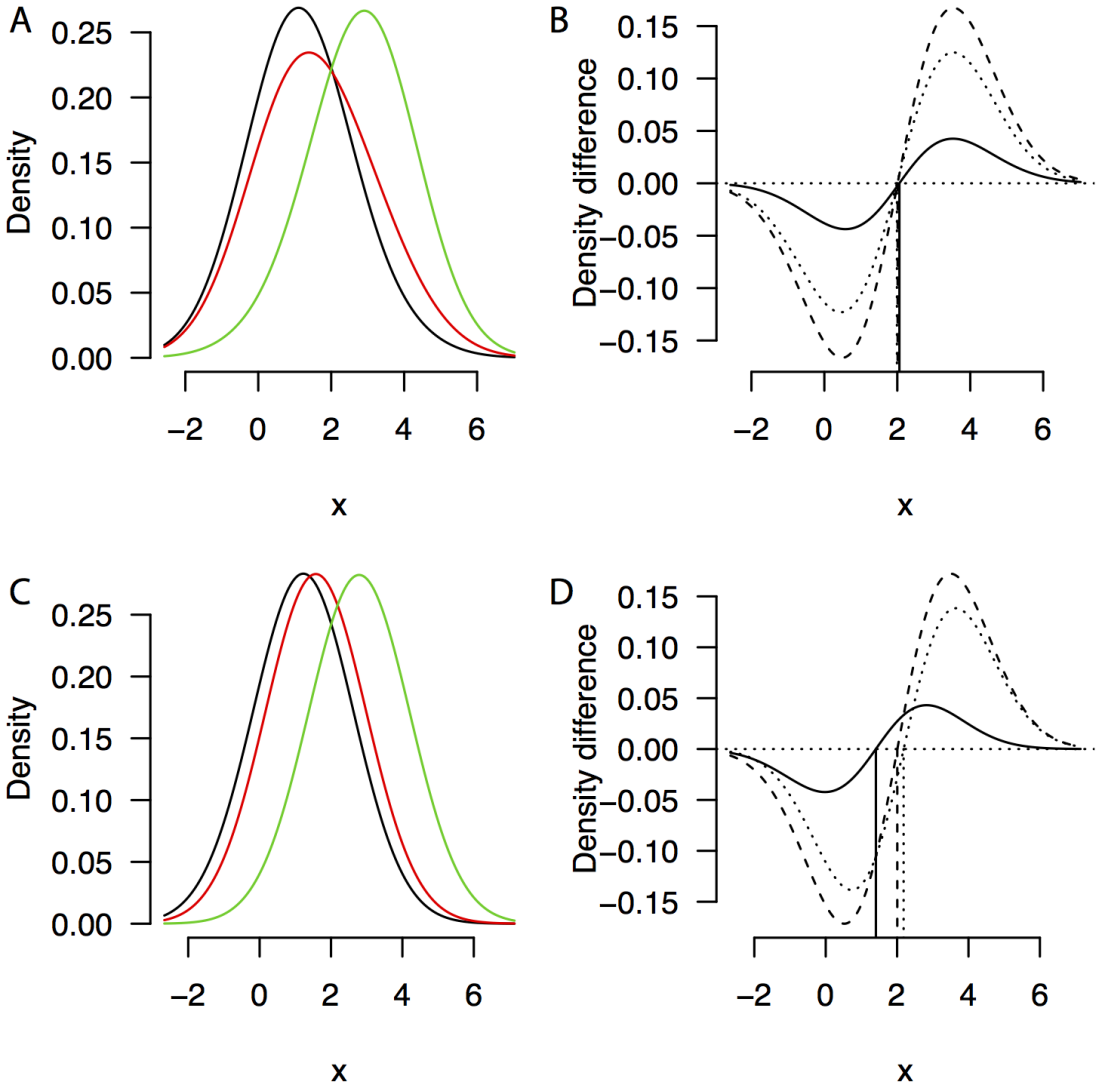
Probability density and density difference for data with and without a fixed-point. Probability density (A, C) and density difference (B, D) for data with (A, B) and without (C, D) a fixed-point. The densities in A correspond to binary mixture distributions with mixture proportions of .1 (black line), .3 (red line), and .9 (green line), respectively. The densities in C correspond to shifted distributions with mean *µ_1_ = 1.2* (black line), *µ_2_ = 1.6* (red line), and *µ_3_ = 2.8* (green line). The solid lines in B and D indicate the difference between the black and red lines in A and C; the dashed lines indicate the difference between the black and green lines; the dotted lines indicate the difference between the red and green lines. The vertical lines in B and D indicate the location of the crossing points.

Density estimation can be used to compute the fixed-point property. Figure 2A shows the density functions of three binary mixture distributions with normally distributed base functions. The means and standard deviations of the base functions are *µ_1_ = 1* and *µ_2_ = 3*, and *σ = 1* for both base functions. The mixture proportions are .1, .3, and .9. As said, the fixed-point property manifests itself as the point where the three density functions intersect (Figure 2A, at *x* = 2). Put differently, this is the x-coordinate where the pairwise differences between the density functions are zero (Figure 2B, at *x* = 2). Each line in Figure 2B represents the difference of two lines in Figure 2A. Thus, three mixture-proportion conditions (1, 2, and 3) result in three pairwise differences (1 vs 2, 2 vs 3, and 1 vs 3). If the fixed-point property holds, then the x-coordinates where the pairwise differences are zero should be equal. We will refer to the points where the difference crosses the x-axis as the *crossing points*. Obviously, to assess whether multiple density functions cross each other at the same x-coordinate, at least three mixture distributions are required resulting in three crossing points.

Figure 2C and 2D illustrate that in the absence of the fixed-point property the crossing points differ. The distributions in Figure 2C are normally distributed with means *µ_1_ = 1.2*, *µ_2_ = 1.6*, and *µ_3_ = 2.8*, with the same standard deviation *σ = 1*. These distributions are thus shifted relative to each other and cannot be considered mixtures from two competing strategies that differ in mixture proportion (cf. [22]). The pairwise density differences show that the crossing points are not aligned at the same x-coordinate (that is, the same RT), an observation that is clear in Figure 2D. Hence, there is no fixed-point property in this data set.

### Testing the fixed-point property

While a graphical demonstration of the fixed-point property may be convincing, inferences from data should ideally be based on the results of sound statistical tests. In our approach, such tests are concerned with assessing the degree to which the binary-mixture hypothesis is supported by the distribution of estimated between-conditions crossing points. In the case of the fixed-point property in RT data, we want to find support for either the hypothesis that the data comes from binary mixture distributions with different mixture proportions (that is, the fixed-point should be observed), or not. Thus, for the fixed-point property to hold, there should be evidence *against* a difference in the crossing points (i.e., no difference should be found between conditions, see Figure 2B). That is to say, there should be evidence in favor of a null hypothesis that there is no difference between crossing-point conditions. Standard null-hypothesis significance tests typically only quantify support against the null hypothesis [23]. Thus, in the absence of a significant effect indicating a difference between the crossing-point conditions, nothing may be said about the equivalence of the conditions, and hence nothing may be said about the presence of the fixed-point property in the data. To solve this problem, we advocate Bayesian hypothesis testing to allow quantification of support for the hypothesis that there is no effect [15]. This way, it can be assessed what the probability is that the fixed-point property holds in the data.

Typically in an experiment, we want to infer whether a certain property exists for the population, based on the sample of participants that were tested. In the current discussion, this means that we want to test whether the fixed-point property holds for the sample of participants in a study. This is the case if we find support for the hypothesis that the crossing points for the various pairs of mixture proportions do not differ. Once the distributions of crossing points per pair of mixture proportion conditions for each of the participants are known, Bayes factors for a regular analysis of variance can be computed [24] to assess the evidence for or against the fixed-point property. A Bayes factor quantifies how much more likely it is that the observed data is generated under one model relative to another model. In this case, it quantifies the probability that the observed distribution of crossing points per condition are generated from one true distribution (H0: the fixed-point property holds) or from multiple distributions (H1: the fixed-point property does not hold as the intersections are not associated with the same RT). Because of the minimum of three conditions and thus three crossing points to assess the fixed-point property in data, it is appropriate to perform a Bayesian ANOVA. Here, we use the standard Bayesian ANOVA implemented in the BayesFactor package in R (http://http://cran.r-project.org/web/packages/BayesFactor), including its standard assumptions with respect to priors. (For a full discussion of this method and its assumptions, see [24]) Obviously, in particular when the null hypothesis is rejected, standard null hypothesis tests may be relevant. In addition to the Bayes factors for the factors in an ANOVA design, the R package associated with this paper – called *fp* for fixed-point – provides conventional F statistics and p-values, if desired.

## Simulation studies

To validate the method for computing and testing the fixed-point property, we ran a series of Monte Carlo simulations. Simulation 1 illustrates that our approach produces reasonable results for non-Gaussian distribution functions, as are typically observed in RT data (e.g., [25]–[27]) and is robust against mild random effects in the data. In Simulation 2 we extend this result to illustrate how the method depends on the mean and standard deviation of the base distributions. In particular, we show that the fp method is capable of distinguishing between the case of true mixture distributions and plausible alternative hypotheses, even when the base distributions exhibit considerable overlap. Simulations 3 and 4 study the effects of sample size and the number of observations, as these influence the power of the test on the one hand, but the precision of the estimate - potentially increasing the chance of finding a difference - on the other hand. In addition, as discussed in the introduction, we assess the influence of the smoothing parameter in the Gaussian kernel density estimation.

### Simulation 1: Robustness against random fluctuations

**Figure 3:**
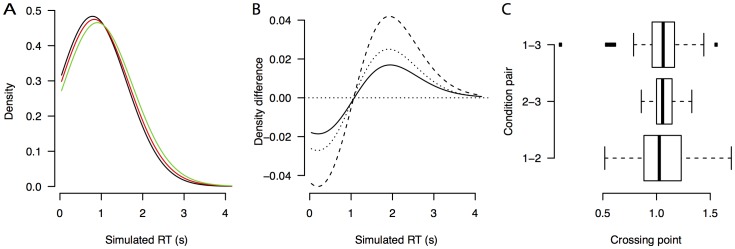
Averaged density, density differences, and crossing points for Simulation 1. Averaged density (A), density difference curves (B), and boxplots for the distributions of crossing points (C) for the data from Simulation 1.

In Simulation 1, we assume observations are sampled from one of two inverse Gaussian base distributions, with scale *λ* = 5 and mean *µ_1_* = 0.8 and *µ_2_* = 1.0 respectively. The inverse Gaussian distribution is an often-used approximation of response time distributions, in particular in situations where only one response alternative is likely or possible (e.g., simple RT tasks [28] or go/no-go tasks [27], [29]). In this simulation, the mixture proportions are arbitrarily set at .1, .4, and .8, indicating that it is more likely to sample from the first (*p* = .8, Strategy 1 is more likely than Strategy 2) or the second (*p* = .1, Strategy 2 is more likely than Strategy 1) distribution, or that both distributions are about equally likely, with a slight tendency towards Strategy 2 (*p* = .4). For each simulated participant, we sampled 200 observations per mixture condition by randomly drawing from the base distributions according to the mixture probabilities. This procedure entails that although the mixture proportions are equal for each participant, the number of observations from each base distributions is not necessarily equal. We simulated 50 participants, adding a normally distributed random effect with a standard deviation of *σ* = 0.1. Figure 3A and B summarize these data by showing the estimated densities and density difference curves across all observations, ignoring the random effects structure in the data. The figures suggest the presence of a fixed point. Using the fp package in R, we computed density difference curves and crossing points for each simulated participant. The distribution of crossing points for the three mixture conditions is presented in Figure 3C. A Bayesian ANOVA gives a Bayes factor in favor of the alternative hypothesis that these three conditions differ of BF_01_ = 0.098, which means it is 10.2 times more likely that there is a fixed point in the data than that there is no fixed point (Not surprisingly, standard frequentist statistics show no support for the alternative hypothesis, F(2,49)  = 0.40, p = .67). This means that there is reason to accept the null hypothesis that there is a fixed point.

### Simulation 2: Effect size

A crucial question is to what extend the method to detect mixture distributions described here depends on the nature of the base distributions. Clearly, if the base distributions have a large difference in means relative to their standard deviations, the mixtures will show signs of bimodality. In contrast, when the means of the base distributions are very similar, it might not be possible to distinguish binary mixture distributions from non-mixture distributions. In Simulation 2, we generated data from two inverse Gaussian distributions with different means and scale parameters. One base distribution was always fixed with with *µ_1_* = 200 and scale *λ_1_* = 100. The mean of the other base distribution was set at a value in the range *µ_2_* = {225;1175}, with a scale set at *λ_2_* = *λ_1_*+*µ_2_*- *µ_1_*. This way, the standard deviation of the second base distribution increases approximately linearly with the mean, similar to what is often observed in response time data [30]. In this simulation, the mixture proportions are 0.0, .5, and 1.0, indicating that either one of the base distributions contributes to the crossing points, or a 50/50 mixture. We simulated data for 50 participants, with 200 observations per condition as before. This number seems a reasonable representation of a real-life data set. In Simulations 3 and 4 we explore the extend to which our method is susceptible to variations of sample size and the number of observations.

In addition to the mixture data, we also simulated data in which the three observed distributions were shifted relative to each other (cf. [22]). A shifted distribution yields the same mean response times, but differences in the shape of the distribution relative to mixture data. In this simulation, the means of the three observed distributions were set at *µ_1_*, (*µ_1_*+*µ_2_*)/2, and *µ_2_*, identical to the mixture data. Similarly, the scales were set at *λ_1_*, (*λ_1_*+*λ_2_*)/2, and *λ_2_*. Finally, the SD of the smoothing kernel was set at 100, a value that balances oversmoothing and overestimation of the density function.

**Figure 4:**
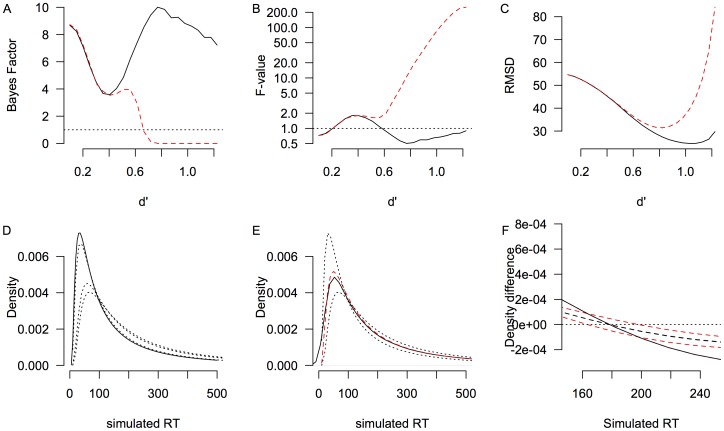
The range of base distributions for which the fixed-point property can be computed (Simulation 2). (A) Bayes factors for mixture data (solid black line) and shifted data (dashed red line). (B) F-values for mixture data (solid black line) and shifted data (dashed red line). (C) The average differences between the crossing points for mixture data (solid black lines) and shifted data (dashed red lines). (D) Base distributions of Simulation 2. Solid line represents Process 1, dashed lines represent alternatives of Process 2. In particular, the dashed lines represent the smallest *d'*, the largest *d'*, and the smallest *d'* for which the BF of the shifted distribution is larger than 1. (E) .5 mixture distribution (solid black line) and the middle shifted distribution (dashed red line) for the smallest *d'* for which the BF of the shifted distribution is larger than 1. For reference, the base distributions are also displayed (dotted lines). (F) Density differences of the three observed mixture distributions (black lines) and the three observed shift distributions for the smallest *d'* for which the BF of the shifted distribution is larger than 1. The solid black line represents the density difference between the base distributions, which is equal for the mixture and shift data. Because the mixture proportion is .5, the density differences of the base distributions with the third distribution are equal and the dashed black line represents both.

Figure 4 displays the results of 4,500 simulated data sets. Figures 4A and B display the Bayes factors and F values for each data set, as a function of the difference between the base distributions, expressed as *d′*. Even for moderately small *d′* values the method correctly distinguishes between mixed distributions and shifted distributions. That is, for this simulation, after about *d′ = 0.4* the BFs of the two types of distributions diverge. The difference between these hypotheses (mixed and shift) can be quantified by computing a likelihood ratio [22]. The likelihood ratio will provide an indication of the likelihood of the fixed-point property relative to another hypothesis, such as the shift-hypothesis. In the absence of a suitable alternative hypothesis, the Bayes factor of the tested data gives a reasonable estimate of the likelihood that the fixed-point property is present.

As an illustration of the size of the effects that the fp method detects, consider the example base distributions in Figure 4D. The dashed lines represent the smallest and largest *d′* value in the simulation (*d′ = 0.1* and *d′ = 1.2*, respectively), as well as the smallest *d′* for which the method indicates that the shifted data set has a Bayes factor smaller than 1. A Bayes factor between zero and 1 indicates support for the null hypothesis, which in the current discussion means support for a fixed-point property. It is clear that a mixture of these distributions would not lead to obvious bimodality in the data (Figure 4E), which calls for a test like the one discussed here. Figure 4F shows the density differences for the smallest *d′* for which the method indicates that the shifted data set has a BF <1. The density differences between the pairs of mixture distributions are indicated by the black solid and black dashed lines. There is only one black dashed line visible because in this simulation two of the three density differences completely overlap. This reflects the choice of a mixture proportion of .5, which results in a mixture distribution that differs equally from both base distributions. The red dashed lines indicate the density differences for the shifted data set. Figure 4F clearly shows that the crossing points of the shifted data sets differ (i.e., the simulated RT at which the density differences are 0 differs). For the mixed data set, the crossing points are identical (the simulated RT at which the density differences are 0 is the same).

### Simulation 3: Sample size

Because both the power of a study as well as the type I error rate depend on the sample size, we explored the impact of sample size on the probability of finding the fixed-point property. To achieve this, we simulated data from varying numbers of participants (Simulation 3), as well as from varying numbers of observations (Simulation 4). This way, both the sample size (the number of participants) and the precision of the fixed point estimate (based on the number of observations) can be considered. In the next sections, we varied the width of the smoothing kernel used for density estimation to study how this impacts the test statistics.

**Figure 5:**
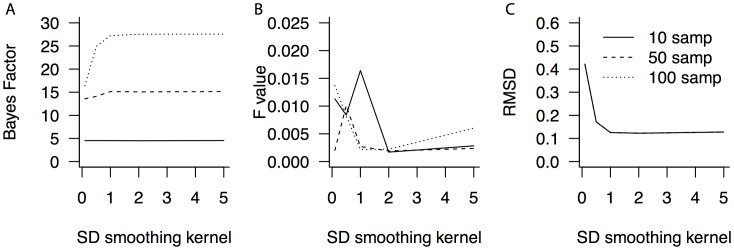
Bayes factors, F statistics and precision as a function of sample size and kernel width. Bayes factors (A) and F statistics (B) differ with sample size (lines) and the standard deviation of the Gaussian kernel. (C) The precision of the estimated crossing points does not vary with sample size. samp: sample size (i.e., the number of participants).

We simulated data for either 10, 50, or 100 participants, with 200 observations per condition. In this simulation we assumed two normally distributed base distributions, with *µ_1_ = 0* and *µ_2_ = 1.5*, and an equal standard deviation of *σ = 1.* The mixture proportions were .1, .5, and .9. We performed Bayesian ANOVAs to assess the evidence for the fixed-point property and compute standard repeated measures ANOVAs. This was repeated 10,000 times to obtain a stable estimate of the Bayes factor and the F statistic. Figure 5 presents the results of Simulation 3. Figure 5A presents the mode of the Bayes factor in favor of the null hypothesis; Figure 5B presents the mode of the F value from the frequentist analysis; Figure 5C presents the root mean squared deviation (RMSD) of the crossing points, indicating how precise these are estimated. Clearly, both Bayes factors and F values are not affected by the width of the smoothing kernel above a reasonable lower bound of roughly *h = *1 SD. However, as the sample size increases, the Bayes factors become more extreme. This can be seen by the different lines, indicating different numbers of simulated participants. Thus, a larger sample size means more confidence in the inference that a fixed-point is present. Still, even for as few as 10 participants, the method can still reliably infer the fixed-point property, with an averaged Bayes factor in favor of the null hypothesis of 4.5.

Figure 5C shows that the sample size does not affect this average precision of the crossing-point estimates, as the lines for different sample sizes overlap. This is because the precision of the crossing-point estimates is crucially determined by the number of observations per participant, as the number of observations is what determines how reliable the density function is estimated.

### Simulation 4: Number of observations

**Figure 6:**
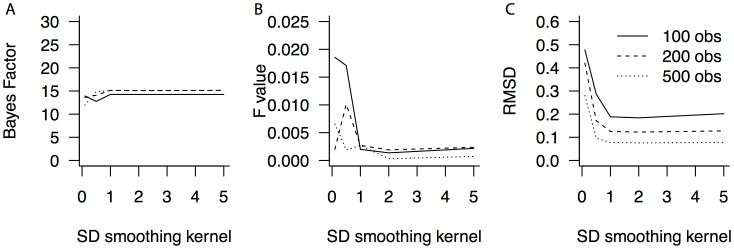
Bayes factors, F statistics and precision as a function of the number of observations and kernel width. Bayes factors (A) and F statistics (B) differ with the number of observations (lines) and the standard deviation of the Gaussian kernel. (C) The precision of the estimated crossing points varies with the number of observations. obs: the number of observations (i.e., repeated measures).

Simulation 4 was set up in a similar way as Simulation 3. That is, again 10,000 simulations were performed, while generating data from distributions with the same properties. The difference lies in the ratio between the number of samples and the number of observations. The number of samples in Simulation 4 was kept constant at 50, while the number of observations per condition varied from 100, to 200, to 500. Figure 6 presents the results of Simulation 4, in which the number of observations per condition is varied. Similar to Simulation 3, the standard deviation of the Gaussian kernel does not influence the results above a lower bound of approximately *h* = 1 SD. A limited set of observations leads to a larger error in estimating the crossing points (Figure 6C), which in turn results in Bayes factors and F statistics that represent greater uncertainty (Figure 6A–B), although these differences are minor.

## Application: The fixed-point property in task switching

As an illustration of how the fp package can easily be applied to test the prediction that a binary mixture distribution underlies the data, we studied the “failure-to-engage” hypothesis of task switching (FTE, [3]). Task switching typically involves two or more tasks that alternate in a sequence of trials, such that participants either have to perform the same task on consecutive trials (the second trial is referred to as a non-switch trial) or perform different tasks on consecutive trials (i.e., switch trials). Numerous studies have shown that switching between tasks involves a cost in terms of increased response times for switch trials relative to non-switch trials (e.g., [3], [31], [32]). To some extent, this effect remains even if the upcoming task is known in advance and there is ample time to prepare. This effect is referred to as the “residual switch cost” [32].

The FTE hypothesis explains residual switch costs by proposing that task preparation only occurs on a subset of trials. That is, on some trials participants fail to prepare for the new task, leading to additional time costs when executing the task. Formally, the FTE hypothesis thus proposes that the RT distribution of switch trials is
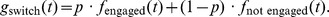



Here, *p* refers to the proportion of trials on which participants fail to prepare and *f*_engaged_ and *f*_not engaged_ refer to the RT distributions of prepared and not prepared trials, respectively.

### Methods

De Jong ([3], Experiment 2) asked 20 participants to perform two tasks sequentially. The sequence was such that there was always a task repetition followed by a task switch (i.e., an RRSSRRSS sequence). Thus, participants knew in advance whether a task switch would occur. There were two manipulations in the experiment that are important for our current purposes: (1) There was a variable response to stimulus interval (RSI) that could be either short (150 ms), medium (600 ms), or long (1500 ms). The rationale was that this manipulation allowed less or more task preparation on switch trials. In terms of the FTE hypothesis, this should influence the mixture proportion *p*. Here, following De Jong [3], we compared the non-switch trials with the long RSI, the switch trials with the long RSI, and the switch trials with the short RSI. (2) Half of the subjects received short blocks (100 blocks of 12 trials), whereas the other half of the participants received long blocks (12 blocks of 96 trials). De Jong [3] argued that the block duration should affect the proportion of trials on which participants fail to prepare, due to the mental effort associated with maintaining the task sequence [33]. Again, this should influence the mixture proportion *p*. We refer to De Jong [3] for a detailed description of the task.

For each participant, we first estimated density functions for each RSI condition, with a smoothing kernel SD of 0.1 s. Next, the difference between these densities was computed as well as the crossing points. (Bayesian) mixed-design ANOVAs are used to infer the presence or absence of the fixed-point property. In particular, a mixed-design ANOVA model was fit to the data with block as a between-subject factor and RSI as a within-subject factor. Next, the fit of this model against a model that omits each factor separately results in a Bayes factor indicating the likelihood that a particular factor is required to explain the data [24]. To place these results into the perspective of Simulation 2, we also computed the average *d′* across participants, under the assumption that the non-switch trials with the long RSI and the switch trials with the short RSI constitute the base distributions comprising the mixture [3].

### Results

**Figure 7:**
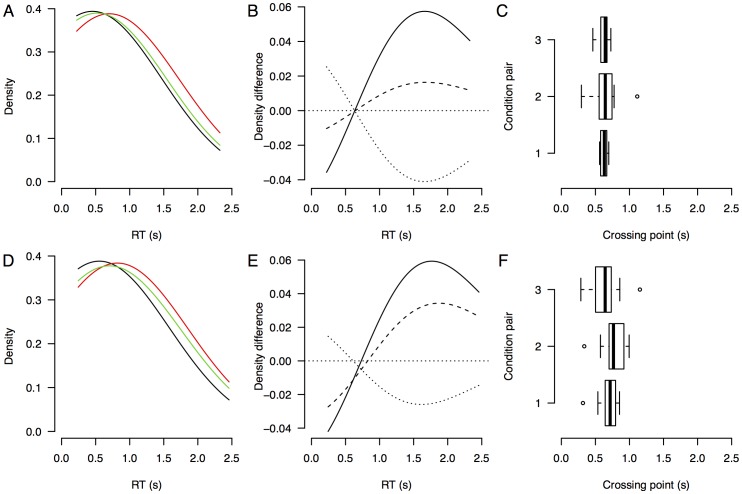
Averaged density, density differences, and crossing points for De Jong (2000), Experiment 2. Averaged density (A, D) density difference curves (B, E), and boxplots for the distributions of crossing points (C, F) for the data from De Jong (2000), Experiment 2. The top row (A, B, C) shows the short blocks, the bottom row (D, E, F) shows the long blocks.

The FTE hypothesis predicts that there exists a fixed-point in the data. In particular, the RT distributions of the three different RSI conditions that we compared should have a common fixed point, as well as the RSI conditions across the between-subject block duration manipulation. Figure 7 visualizes that indeed the fixed point property holds in this data set. A Bayesian mixed-effects ANOVA shows that the Bayes factors of the main effect of RSI were BF_RSI_ = 0.29 (the data is 3.4 times more likely under the null hypothesis than under a model that includes RSI as a factor) and BF_RSI × block_ = 0.27 (the data is is 3.7 times more likely under a model without the interaction – but with main effects – than under the full model with RSI, block and the interaction). A classical mixed-effect ANOVA with block length as between-subjects factor and RSI as within-subjects factor indeed does not find support for the alternative hypothesis (F_RSI_(2,36) = 0.78, p = 0.47, F_RSI × block_(2,36) = 0.28, p = 0.76). In addition, there was no clear effect of the block duration (BF_block_ = 0.51), suggesting that the data is only 2.0 times more likely to come from a model without block duration than with block duration (A standard frequentist test yields F_block_(1,18) = 2.8, p = 0.11).

The average *d′* for the short blocks was 1.30 (SE = 0.093) and the average *d′* for the long blocks was 1.09 (SE = 0.16). For both block durations, the average *d′* is in the range for which a high BF in favor of the fixed-point property is indeed an indicator of binary mixture data, rather than alternative hypotheses such as shifted data.

### Discussion

The results of the fixed-point analysis on the data of Experiment 2 of De Jong [3] generally support the FTE hypothesis. That is, the prediction that the different RSI and block durations have different mixture proportions of the RT distribution of switch trials is supported because we confirmed that the fixed-point property holds in the data. The finding that the crossing points differed between the block duration groups could be due to randomization failures, or general processing differences in the two groups that are unrelated to the mixture proportion. However, if the fixed-point property would have been confirmed in one group but not the other, then the RSI × Block duration interaction would have been significant, and the Bayes factor of the full model against the model that omitted the interaction would have been larger then 1. Therefore, for both block duration conditions there is considerable support in favor of the fixed-point property. As the FTE hypothesis predicts this specific and nontrivial property, these results support the FTE hypothesis.

## General Discussion

The fixed-point property in binary mixture data is an interesting prediction for many theories in cognitive psychology that assume mixtures of processes. If the mixture proportions are experimentally manipulated, then it can be easily verified whether the fixed-point property holds in the data. This paper has outlined how this can be achieved. Accompanying this paper is an R package called *fp* that implements the computation and test of the fixed-point property. The package can be retrieved from http://www.leendertvanmaanen.com/fp, and is available as supporting information with this article.

In a series of simulations, we tested the method proposed here as well as the R package, and found that it can successfully distinguish between data sets from binary mixture distributions and data sets with other but comparable differences in RT. In particular, we tested the method on a data set in which three distributions were shifted relative to each other (instead of mixed), and found that for large enough *d′* values, the fp method found evidence against the fixed-point property. If a shift in the data is a reasonable hypothesis, then the fp method can be used to compute a likelihood ratio. In this case, other methods to distinghuish between mixture data and shifted data become available as well [22], [34]. However, in the absence of a specific alternative hypothesis, the fp test provides the likelihood of a fixed-point property in the data, which can be indicative of binary mixture distributions.

Furthermore, the test is robust against variations in the Gaussian kernel standard deviation, which determines the smoothness of the estimates density functions. When the standard deviation of the kernel was set at a suitably high value exceeding one standard deviation, the results remained comparable. However, there is a practical limit on increasing the kernel SD. If the SD is too large, the density estimate oversmoothes important properties of the RT distribution related to bimodality. The test is also reasonably robust against low number of observations and small sample sizes such that it can be applied to relatively small data sets.

Finally, to show the applicability of the fixed-point property test, we analyzed data from De Jong [3]. The data was collected to support the FTE hypothesis, which assumes that response time distributions of task switch trials are a binary mixture of trials on which participants prepare for the upcoming task, and trials on which they fail to prepare. The two experimental manipulations in the experiment were aimed at changing the mixture proportion, making the data suitable for studying the fixed-point property. The results of our fixed-point analyses align with De Jong's [3] original conclusions, and are in support of the FTE hypothesis.

These simulations and analysis of an existing data set demonstrate that the fixed-point property, and the *fp package*, can be a valuable tool in the statistical toolbox of cognitive (neuro-) scientists.

## Supporting Information

R code S1 **Downloadable fp package.**
(GZ) Click here for additional data file.
